# Plasma biomarkers of vascular injury and cerebrovascular reactivity in *APOE4* carriers

**DOI:** 10.1002/alz.087589

**Published:** 2025-01-03

**Authors:** Arunima Kapoor, Shubir Dutt, Amy Nguyen, Aimée Gaubert, John Paul M Alitin, Isabel Sible, Anisa J Marshall, Fatemah Shenasa, Allison C Engstrom, Xingfeng Shao, David Bradford, Kathleen E. Rodgers, Danny JJ Wang, Daniel A. Nation

**Affiliations:** ^1^ University of California, Irvine, Irvine, CA USA; ^2^ University of Southern California, Los Angeles, CA USA; ^3^ University of Arizona, Tucson, AZ USA

## Abstract

**Background:**

*APOE4* carriers exhibit cerebrovascular dysfunction which may contribute to the development of cognitive decline and dementia; however, the mechanisms underlying this pathophysiology remain unknown. Impaired cerebrovascular reactivity (CVR) may be associated with vascular injury, inflammation, and endothelial dysfunction. To examine whether these processes may be involved in CVR deficits in *APOE4* carriers, we explored whether plasma levels of vascular injury markers indicative of inflammation and endothelial dysfunction are associated with impaired CVR to hypercapnia and hypocapnia in older *APOE4* carriers.

**Method:**

Sixty‐five independently living older adults (mean age = 69.3 years; SD = 8.6; age range 55‐89 years; 30.8% male) free of dementia or clinical stroke were recruited from the community and underwent venipuncture and brain MRI. Plasma was assayed for vascular injury markers (Serum amyloid A (SAA), C‐reactive protein (CRP), intercellular adhesion molecule 1 (ICAM‐1), vascular cell adhesion molecule 1 (VCAM‐1)) and values were log‐transformed. *APOE* genotyping was conducted on blood samples. Pseudo‐continuous arterial spin labeling MRI quantified whole brain cerebral perfusion during CVR to hypocapnia (0.1Hz paced breathing; vasoconstriction).

**Result:**

Adjusting for age and sex, multiple linear regression analysis revealed a significant association between whole brain CVR to hypocapnia and circulating levels of SAA, VCAM‐1 and ICAM‐1. Analyses stratified by *APOE4* status revealed a significant association between whole brain CVR to hypocapnia and levels of SAA [B = ‐5.39, 95% CI (‐9.78, ‐1.01), p = .018], VCAM‐1 [B = ‐10.70, 95% CI (‐17.59, ‐3.82), p = .004] and ICAM‐1 [B = ‐10.95, 95% CI (‐17.44, ‐4.46), p = .002] in *APOE4* carriers but not non‐carriers.

**Conclusion:**

Findings suggest that elevated levels of vascular injury markers are associated with deficits in cerebral vasoconstriction, particularly in *APOE4* carriers at genetic risk for Alzheimer’s disease. SAA, VCAM‐1 and ICAM‐1 are known to be involved in inflammation and endothelial dysfunction, and elevated levels are associated with cerebrovascular resistance and cognitive impairment. Our findings suggest that *APOE4* carriers may be particularly vulnerable to these pathophysiological processes. Further longitudinal studies may elucidate whether inflammation and endothelial dysfunction precede or precipitate CVR deficits and contribute to cognitive decline in *APOE4* carriers.

